# Colorism and eating disorders (ED) among BIPOC: expanding the skin tone trauma model to conceptualize a pathway

**DOI:** 10.3389/fpsyt.2026.1811526

**Published:** 2026-06-02

**Authors:** Ashley Acle

**Affiliations:** Private Practitioner, San Francisco, CA, United States

**Keywords:** BIPOC, Black, colorism, eating disorders – classification, Indigenous and people of color, skin tone trauma

## 1 Introduction

Despite extant research suggesting eating disorders (ED) prevalence across minoritized racial and ethnic groups (BIPOC, including Black, Indigenous, Asian, Latine, Multiracial/Multiethnic People of Color), racial disparities persist in diagnosis, referral and treatment, with BIPOC seeking and receiving ED treatment less than Whites (1–3). Risk factors for ED among BIPOC may differ from White peers’ (e.g., acculturative stress, 4, 5), as well as clinical presentations and culturally specific body image ideals (e.g., idealization of specific facial features, greater acceptance for larger body sizes, preference for muscle definition or curves in specific body parts). Addressing culture is imperative in ED, particularly in treatment, yet severely lagging relative to other areas of psychology and counseling (6). A critical gap remains in understanding ED among BIPOC, culturally affirming diagnosis and treatment, and relevant cultural considerations.

One underdiscussed consideration relevant to ED and body image among BIPOC is colorism. Colorism, discrimination based on the lightness or darkness of one’s skin tone, has negative impacts psychologically, emotionally, physically, and socially. Colorism is distinct yet similar to racism and occurs worldwide, across the skin color spectrum, through comments, societal messages, (in)opportunities and general treatment, with disadvantages toward individuals with darker skin tones outweighing those with light skin. Per Sissoko et al. (7), colorism is conceptualized as a “colonial tool and a psychological consequence of White Supremacy”.

## 2 Colorist incidents and skin tone trauma: potential pathway to ED

Experiencing colorism, or colorist incidents, like other forms of interpersonal and systemic trauma, are largely out of one’s control. These may be overt, covert, intentional, unintentional, single or repetitive events, and experienced through language and/or symbols (8). Colorist incidents also occur through institutions (e.g., individuals with darker skin face harsher punishments, poorer healthcare and responsiveness to their pain, less educational support). Colorist incidents have cumulative and adverse effects on health, occur across the lifespan, and can occur within one’s racial group (ingroup), within other minoritized racial groups, and within White groups.

One hypothetical pathway from colorist incidents to ED is via skin tone trauma. Skin tone trauma, a conceptual framework originally proposed by Landor and McNeil Smith (8) to understand the psychological, emotional, physical, relational and behavioral vulnerability that may result from experiencing colorism, can have negative effects on one’s sense of self, identity development, body satisfaction and interpersonal relationships (8). ED may arise as a means of coping with colorist incidents and skin tone trauma, though this has not been explored in the literature.

## 3 Proposed conceptual model: Colorism, Skin Tone Trauma, and ED among BIPOC

**Figure 1:**
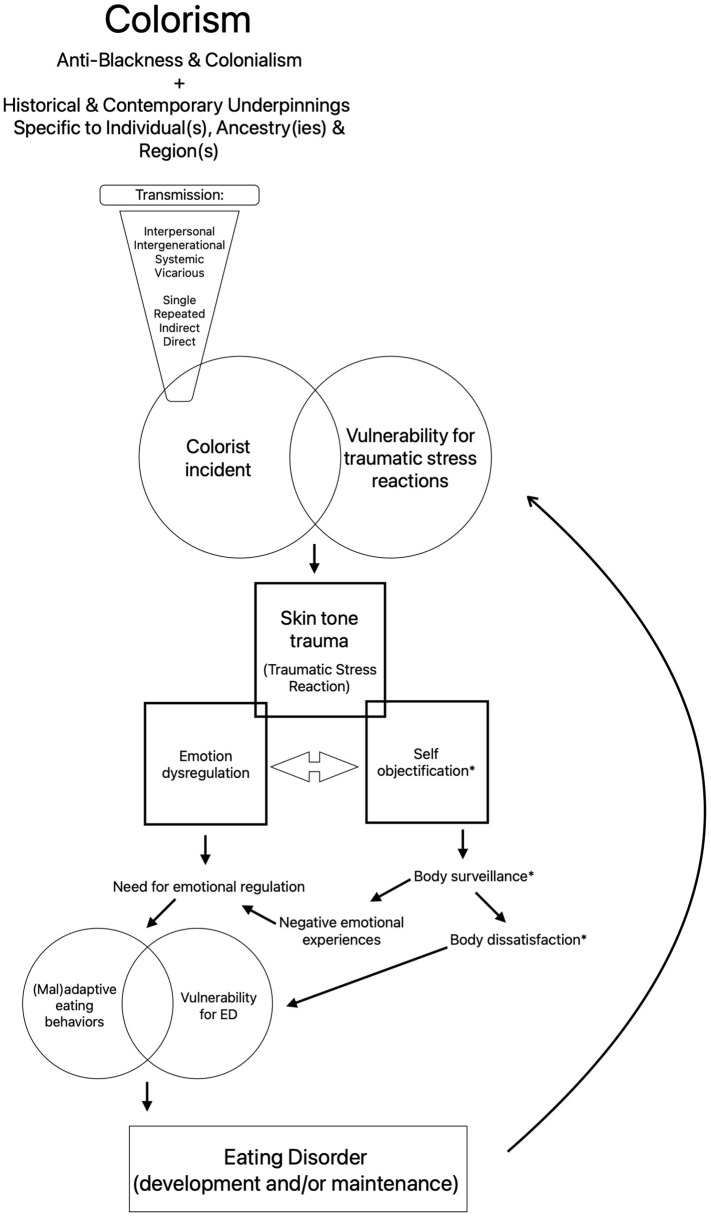
Colorism, Skin Tone Trauma and Eating Disorders (ED) model.

The Colorism, Skin Tone Trauma, and ED model (**Figure 1**) expands upon Landor and McNeil Smith’s (8) model, with additional attention to the underpinnings of colorism specific to the individual and consideration of vulnerability models, intersectionality, emotional dysregulation and self-objectification. This model notes that ED may alter future vulnerability to colorism.

### 3.1 Historical underpinnings of colorism relevant to the individual(s), ancestry(ies) and region(s)

In applying this framework across BIPOC, this model proposes consideration of the historical and contemporary underpinnings of anti-blackness, the transatlantic slave trade, and colonialism from the original mode, as well as the pre-colonial underpinnings, specific history, and contemporary experiences in the country(ies)/region(s) of ancestry specific to the individual(s) served.

Colorism’s underpinnings are nuanced across regions/countries. For example, colorism pre-dates European colonialism in parts of the world, like China and Japan, where light skin was associated with higher status class and aesthetics in as early as 700s writing and art (9–11). Colonialism cemented the link between status and skin color through the institutionalization of classism and further anti-blackness, reinforcement through violence and deprivation, and an increasing mixing of people (e.g., India; 9). Contextualizing contemporary experiences of colorism within more recent history (e.g., multiple periods of colonial rule, sociopolitical changes, occupation) and globalization may also be relevant to a nuanced understanding of colorism’s underpinnings, particularly intersecting facets of colorism (e.g., gendered colorism and beauty ideals, biracial/multiracial colorism, colorism and immigration).

### 3.2 Additional consideration of vulnerabilities (individual experiences and intersectionality)

ED are multidetermined, influenced by a host of risk factors across biology, psychology, genetics and epigenetics, culture, and environment. Traumatic stress reactions are also complex: individual factors, environment, and characteristics of the traumatic exposure influence symptom development and severity. Vulnerability models in the proposed framework integrate these considerations. As vulnerability is dynamic, especially as it relates to social influences and continuous identity development, these models also encapsulate one’s intersecting identities and how they are shaped by constructs like sizeism, ableism, citizenship/immigration status, stable access to basic resources (i.e., housing, food and water security, transportation), along with the original intersectionality considerations.

### 3.3 Traumatic stress reactions, emotion dysregulation, and self-objectification: paths toward ED

This conceptual model draws attention to the processes of emotional dysregulation and self-objectification stemming from skin tone trauma as they may relate to ED. Of note, these two paths may be both independent and influence each other. It is also possible that the pathway between skin tone trauma and ED differ based on symptomatology, which is a question worth researching.

#### 3.3.1 Skin tone trauma-related emotion dysregulation and ED

The relationship between interpersonal traumatic stress reactions, binge eating, and emotion regulation difficulties is an illustrated connection in the literature (e.g., 12), with additional studies demonstrating significant relationships between trauma exposure and binge eating among BIPOC (e.g., 13). Escape theory (14) and affect regulation theory (15) are the most commonly cited theoretical models relevant to binge eating. Binge eating may present across multiple ED (e.g., BED, BN, AN binge/purge type) to distract and soothe negative affect. Escape theory, or escaping one’s negative views of self and concern over others’ perceptions, may be relevant to the connection between skin tone trauma and ED given the pervasiveness of colorism. Additional research is needed to test this hypothesis.

#### 3.3.2 Skin tone trauma-related self-objectification and ED

Ruminating on appearance from an outside perspective (self-objectification) may offer predictability and distance from self after colorist incidents. Self-objectification as an ED pathway, based on objectification theory (16) and the overall strong implications of self-objectification in the development of ED (e.g., 17), may offer a framework for considering skin-tone related surveillance, body dissatisfaction and the role of (White) thin internalization. Body dissatisfaction is a known risk factor for ED and men and women of color who have experienced colorism report lower rates of skin shade satisfaction, more skin shade surveillance, and greater levels of psychological distress (18). This pathway may also be relevant given links between racial trauma and self-objectification, body surveillance, and body shame (e.g., among Asian Americans, see 19). Future research on whether this pathway may be more relevant for restrictive presentations (e.g. AN) and control is warranted.

## 4 Discussion: implications for research and practice

### 4.1 Assessing for skin tone trauma within a culturally flexible diagnostic approach

Assessing for colorist incidents and skin tone trauma may be important considerations in ED and related body image difficulties. Using a culturally flexible diagnostic approach, i.e. a culturally adaptive, polythetic approach that accommodates cultural factors in reasons for behaviors and a culturally relevant treatment consideration (6), can facilitate beginning with the patient’s own discourse in understanding their experiences of body and skin satisfaction, eating behaviors, and ingroup and outgroup experiences of colorism.

A culturally flexible diagnostic approach to intake may look like establishing a trauma-informed care foundation (e.g., orienting the individual(s) to the process), naming an intention to understand their identities and experiences as they intersect with seeking treatment, and asking open ended questions to explore these (broaching, see 20). Building upon this, providers psychoeducate about skin tone trauma and racial trauma as factors relevant to health, with the consent of the individual(s). A thorough evaluation of trauma, including other systemic, nonpersonal, and interpersonal traumas, should also be completed. This can further elicit dialogue about the meaning of one’s eating behaviors, contextualize experiences, and address psychological and interpersonal maintaining factors.

#### 4.1.1 Validated assessments

Given the proposed pathways, validated measures of colorism (e.g., The Everyday Colourism Scale, Craddock et al. (21) which assesses perceived experiences of colorism) and skin tone satisfaction (e.g., Skin Color Satisfaction Scale, 22 which assess skin color shade satisfaction, self-perceived skin color and ideal skin color among African American women and can be modified for other minoritized racial/ethnic groups and genders) may facilitate assessing and discussing patients’ experiences related to skin color. These assessments, respectively, may be integrated into clinical conversations about social interactions and trauma and assessments of body image, as part of a comprehensive ED evaluation. Assessment results may illuminate whether ED behaviors are tied to skin tone trauma, self-objectification, body dissatisfaction or a combination, and guide treatment conceptualization and intervention.

### 4.2 Considerations for practitioners

To address skin tone trauma, practitioners are required to have a better understanding of culture, including the historical and contemporary influences of oppressive structures (e.g., colorism), patients’ identity (e.g., race, ethnicity, skin color, gender, sexual orientation, immigration/documentation status, ability, size, class, etc.), and how these intersect in the patient’s lived experience and psychological functioning. This coincides with Landor and McNeil Smith’s (8) recommendation that practitioners be educated and trained in concepts like colorism, colorist incidents, and skin tone trauma to engage in meaningful conversations on colorism’s implications and intersections with identity, power, privilege and oppression. This also aligns with bottom up approaches and developing a shared language, validating patients’ experiences and worldviews shaped by colorism and oppressive systems, and exploring early and formative messages about appearance and skin tone (23).

#### 4.2.1 Interventions

Interventions should be implemented to address the combination of colorism, skin tone trauma, and ED. Perspective taking and mindfulness based interventions may be promising for facilitating self-compassionate statements and pride in skin tone (24) and coincide with Acceptance and Commitment Therapy (ACT) approaches that promote adaptive coping and psychological flexibility, specifically a conscious meaningful engagement with one’s values in the midst of painful experiences (25). Clinical approaches that highlight patients’ strengths around skin tone and their other identities can also promote resiliency and healing (26, 27). If using evidence-based treatments, culturally adapted interventions are beneficial (28). Given the transdiagnostic characteristic of emotion dysregulation across ED, interventions focused on this feature and based in critical race psychology (e.g., antiracist adaptations to Dialectical Behavior Therapy, DBT, 29) may be most appropriate. Interventions that address racial trauma [e.g., Trauma Focused Cognitive Behavioral Therapy, TF-CBT, and Racial Socialization for Youth of Color, Metzger et al. (30)] may also be relevant for addressing skin tone trauma and colorism. Enhanced Cognitive Behavioral Therapy (CBT-E, 31) may be amenable.

In working with families and communities, interventions should focus on reducing negative interpersonal outcomes (e.g., isolation, poor interpersonal relationships and distrust), provide education to decrease appearance-based comments and promote positive self-identity and pride in skin tone. Interventions addressing stigma and shame are also worth considering; these may be relevant to skin tone trauma and interpersonal outcomes, impact help-seeking for ED and be heightened among BIPOC, and especially BIPOC who are sexual minorities and/or men (32). Group therapy work, community-based conversations that include generalist healthcare providers and community/spiritual leaders, who may be the first points of health contact, and advocacy work may be beneficial for creating safe spaces where colorism is intentionally named and mitigated.

## 5 Conclusion

The proposed Colorism, Skin Tone Trauma, and ED model provides a culturally relevant framework for exploring how colorist incidents may influence ED and related body image issues faced by BIPOC via consideration of vulnerabilities, intersecting identities, emotion regulation and self-objectification. Future research is needed to empirically test this proposed model, explore mechanisms and moderators, and interventions. Additionally, it may be beneficial to further study what factors underlie vulnerability to skin tone trauma and ED that may be meaningful targets for prevention. This model assumes similarities to pathways between interpersonal trauma and ED, yet this may in itself be a limiting representation of the depth of systemic trauma. Additional exploration between systemic traumas and ED are needed across research and clinical practice to truly address the experiences of the global majority.
